# Effects of Ripasudil Hydrochloride Hydrate-Brimonidine Tartrate Fixed-Dose Combination Using Rho Kinase Inhibitor and Alpha2 Adrenergic Receptor Agonist on Aqueous Column in the Episcleral Vein: A Randomized, Double-Masked, Crossover Clinical Trial (ROCK Alpha-Aqua Study)

**DOI:** 10.3390/jcm15082880

**Published:** 2026-04-10

**Authors:** Marie Suzuki, Shogo Arimura, Kentaro Iwasaki, Yusuke Orii, Hiroshi Kakimoto, Ryohei Komori, Shigeo Yamamura, Masaru Inatani

**Affiliations:** 1 Department of Ophthalmology, Faculty of Medical Sciences, University of Fukui, 23-3 Simoaizuki, Matsuoka, Eiheiji, Yoshida, Fukui 910-0017, Japan; marie.ohgoshi.0408@gmail.com (M.S.); leosunshine33@gmail.com (S.A.); kenkentaro0329@yahoo.co.jp (K.I.); orii@g.u-fukui.ac.jp (Y.O.); hrs0214k@gmail.com (H.K.); yanko3122@yahoo.co.jp (R.K.); 2 The Faculty of Pharmaceutical Sciences, Josai International University, Gumyo 1, Togane 283-8555, Japan; syamamura0803@gmail.com

**Keywords:** Rho kinase inhibitor, ripasudil hydrochloride hydrate–brimonidine tartrate fixed-dose combination, conventional aqueous outflow, episcleral vein aqueous column, hemoglobin video imaging, intraocular pressure

## Abstract

**Background/Objectives**: Rho-associated protein kinase inhibitors reduce intraocular pressure (IOP) by enhancing aqueous humor outflow through the trabecular meshwork–Schlemm’s canal pathway. However, it remains unclear whether the fixed-dose combination of ripasudil hydrochloride hydrate and brimonidine tartrate (GLAALPHA) enhances conventional aqueous outflow in vivo. **Methods**: This single-center randomized clinical trial included healthy adult volunteers who received GLAALPHA, a brimonidine tartrate–brinzolamide fixed-dose combination (Ailamide), or brimonidine tartrate monotherapy (Aiphagan) in a crossover sequence. The aqueous column width in the episcleral veins was assessed at baseline and at 2 h (primary outcome) and 8 h using hemoglobin video imaging. **Results**: Among 24 participants, analyses included 23 GLAALPHA-treated eyes, 21 Ailamide-treated eyes, and 22 Aiphagan-treated eyes. Two hours after instillation, the aqueous column width significantly increased from baseline only in the GLAALPHA group (*p* = 0.002). The percent increase in the aqueous column width at 2 h was significantly greater with GLAALPHA than with Ailamide (*p* = 0.039) and not significantly different between GLAALPHA and Aiphagan (*p* = 0.114). At 8 h, the aqueous column width did not differ from the baseline in any groups. **Conclusions**: In healthy adult eyes, GLAALPHA significantly increased the aqueous column width in the episcleral veins 2 h after instillation, indicating enhanced conventional aqueous outflow. These findings provide evidence that GLAALPHA promotes trabecular outflow beyond the effects of brimonidine tartrate-containing comparators and offer mechanistic insights into its action.

## 1. Introduction

Glaucoma is a major cause of irreversible visual loss worldwide and was estimated to have caused blindness in 3.61 million people and moderate-to-severe visual impairment in 4.14 million people in 2020 [1]. Lowering the intraocular pressure (IOP) remains the only evidence-based strategy for preventing the progression of glaucomatous optic neuropathy [2]. Topical anti-glaucoma medications reduce IOP through three principal mechanisms: suppression of aqueous humor production by the ciliary body, enhancement of aqueous outflow through the conventional trabecular meshwork–Schlemm’s canal pathway, and facilitation of uveoscleral outflow [3,4]. Among the currently available eye drops for glaucoma, those primarily targeting the conventional outflow pathway are limited [5,6].

Rho-associated protein kinase (ROCK) inhibitors are unique class of drugs that directly reduce outflow resistance within the trabecular meshwork and distal outflow structures [7,8,9]. At the molecular level, ROCK inhibitors induce the cytoskeletal relaxation of trabecular meshwork cells, reduces extracellular matrix deposition, and increases the permeability of Schlemm’s canal endothelial cells, thereby enhancing aqueous humor drainage via the conventional pathway [10,11,12,13]. Two ROCK inhibitors, ripasudil hydrochloride hydrate and netarsudil, are currently used in clinical practice for glaucoma treatment, both of which have demonstrated IOP-lowering effects that are distinct from those of prostaglandin analogs or aqueous suppressants [14,15,16,17].

In a previous randomized, double-blind, crossover clinical trial, we demonstrated that topical instillation of ripasudil hydrochloride hydrate significantly increased the width of the aqueous column within human episcleral veins compared with that by latanoprost, providing direct in vivo evidence that ROCK inhibition enhances aqueous outflow through the conventional pathway [18]. This study employed hemoglobin video imaging, a noninvasive slit-lamp-based technique that visualizes laminar aqueous flow within episcleral veins by exploiting the optical absorption properties of hemoglobin [19]. Hemoglobin video imaging enables the quantitative assessment of aqueous outflow under physiological conditions and has been validated in previous studies that evaluated conventional outflow enhancement after minimally invasive glaucoma surgery and selective laser trabeculoplasty [20,21]. Collectively, these studies support the utility of hemoglobin video imaging as a functional biomarker of aqueous humor dynamics in conventional outflow system.

A fixed-dose combination of ripasudil hydrochloride hydrate 0.4% and brimonidine tartrate 0.1% (GLAALPHA; GLAALPHA Combination Ophthalmic Solution; Kowa Co., Ltd., Nagoya, Japan) is a novel anti-glaucoma eye drop that combines a ROCK inhibitor with an α2-adrenergic agonist. Owing to this formulation, GLAALPHA simultaneously targets all three IOP-lowering strategies: suppression of aqueous humor production via brimonidine tartrate, enhancement of uveoscleral outflow via brimonidine tartrate, and reduction in outflow resistance within the trabecular meshwork–Schlemm’s canal pathway via ripasudil hydrochloride hydrate [22]. Although brimonidine tartrate alone reduces IOP by decreasing aqueous production and promoting uveoscleral outflow, it does not enhance the conventional outflow [23]. Therefore, it remains unclear whether the addition of ripasudil hydrochloride hydrate to the fixed-dose combination results in a measurable increase in aqueous humor drainage through the conventional pathway, particularly at the level of the episcleral veins, or whether the brimonidine tartrate component modifies this effect through vascular or aqueous suppressive actions.

The purpose of the present study was to determine whether GLAALPHA enhances aqueous humor outflow through the conventional pathway, as assessed by changes in the aqueous column within the episcleral veins, compared to a fixed-dose combination ophthalmic suspension containing brimonidine tartrate 0.1% and brinzolamide 1%, which lacks a conventional outflow-enhancing mechanism, and brimonidine tartrate monotherapy. Using hemoglobin video imaging, we sought to clarify whether GLAALPHA uniquely increased episcleral aqueous outflow beyond the effects attributable to brimonidine tartrate alone, thereby providing mechanistic insights into its IOP-lowering action in human eyes. This randomized, double-blind, crossover clinical trial was designated as the ROCK Alpha-Aqua study.

## 2. Materials and Methods

### 2.1. Study Design and Setting

This investigator-initiated, single-center, randomized, double-blind, three-period crossover clinical trial was conducted at Fukui University Hospital (Fukui, Japan). We compared the effects of different topical anti-glaucoma medications on the aqueous column within the episcleral veins of healthy adult participants.

### 2.2. Ethics and Trial Registration

This study was conducted in accordance with the tenets of the Declaration of Helsinki and complied with the Japanese Clinical Trials Act. The study protocol, informed consent, and related materials were reviewed and approved by the Certified Review Board, University of Fukui (CRB5180014). The trial was registered in the Japan Registry of Clinical Trials (jRCTs051240091) before study initiation. Written informed consent was obtained from all participants before any study-related procedures were performed. Insurance coverage for research-related harm was provided in accordance with the study protocols.

### 2.3. Participants

Healthy adult volunteers aged 18 years or older were eligible for inclusion in the study. The key exclusion criteria included the presence of any ocular disease, history of ocular surgery, current use of ocular medications, pregnancy or lactation, potential pregnancy during the study period, or any condition deemed inappropriate by the investigators. Contact lens users were required to refrain from wearing lenses during the study period, except during washout intervals. All participants underwent screening examinations to confirm their ocular health and the feasibility of episcleral vein imaging. Individuals for whom adequate imaging could not be obtained prior to randomization were excluded.

### 2.4. Randomization and Masking

Eligible participants were assigned a unique study identification number and randomized into one of the six treatment sequences corresponding to all possible permutations of the three study drugs across the three study periods. Randomization was performed using the permuted-block method with an independent research support unit (Medical Research Support Center, Fukui University Hospital). An unmasked study supporter who was not involved in the outcome assessment administered the assigned eye drops to maintain masking. Both the participants and investigators responsible for outcome assessments remained masked throughout the study. Imaging and clinical examinations were conducted in a separate room to preserve the masking integrity.

### 2.5. Interventions and Study Procedures

Three commercially available ophthalmic formulations were evaluated:

1. GLAALPHA;
2. A fixed-dose combination ophthalmic suspension containing brimonidine tartrate 0.1% and brinzolamide 1% (Ailamide; Senju Pharmaceutical Co., Ltd., Osaka, Japan);
3. Brimonidine tartrate 0.1% ophthalmic solution (Aiphagan; Senju Pharmaceutical Co., Ltd., Osaka, Japan).

During the study period, a single drop of the assigned medication was administered to each eye. Assessments were performed at baseline (pre-instillation) and 2 and 8 h after instillation. Each participant received all three study medications in a crossover manner, with a washout period of at least 2 days between the study periods to minimize potential carryover effects. A washout period of 2 days was implemented between each treatment period. Although formal statistical testing for carryover effects was not pre-specified, this study employed a single-instillation crossover design, and based on the known pharmacodynamic profiles and standard twice-daily dosing of the study drugs, the washout duration was considered sufficient to minimize potential carryover effects. In addition, treatment sequence and period were included as fixed effects in the linear mixed-effects model to account for potential residual effects.

### 2.6. Study Eye Selection and Episcleral Vein Imaging Site

The healthy eye for which episcleral vein imaging was feasible was selected, and the right eye was preferentially selected whenever possible. For episcleral vein imaging, the inferonasal quadrant was preferentially chosen because this region allows clear visualization of the aqueous columns [24]. If adequate imaging was not obtained in the inferonasal quadrant, the inferotemporal quadrant was used. If no aqueous column was identifiable in the inferior quadrants, the superior nasal and temporal quadrants were selected. If adequate imaging could not be obtained for the right eye, the contralateral eye was selected. The collected data included age, sex, eye laterality, IOP, and imaging vein location. The IOP was measured using Goldmann applanation tonometry.

### 2.7. Measurement of Aqueous Column Width

The width of the aqueous column in the episcleral veins was measured using hemoglobin video imaging, following the methodology described in our previous study [18]. Briefly, the episcleral veins were visualized using a slit-lamp biomicroscope equipped with a green filter, and video recordings were obtained through a slit-lamp eyepiece using a digital recording device. Recordings were performed at baseline and at 2 and 8 h after the instillation of the study medications.

The captured video files were converted to AVI format and analyzed using ImageJ software (National Institutes of Health, Bethesda, MD, USA; available at https://imagej.net/ij/). To reduce motion artifacts, image stabilization was performed using the StackReg plugin in Fiji/ImageJ (available at https://imagej.net/plugins/stackreg, accessed on 6 April 2026) prior to the analysis. For each recording, still frames were extracted and a line perpendicular to the vessel axis was placed across an episcleral vein containing a clearly identifiable aqueous column. The aqueous column width was quantified in pixels as the distance between two minimum intensity points on the grayscale intensity profile across the vessel lumen corresponding to the boundaries of the aqueous column. Measurements were independently performed by two blinded observers, and the means of the two measurements were used for statistical analysis.

The aqueous humor flow rate was quantified from the video recordings of the episcleral vein by analyzing the movement of erythrocyte patterns within the aqueous column over time using frame-by-frame image analysis, in accordance with previously established methods [20].

### 2.8. Safety and Tolerability Assessment

The participants completed a comfort questionnaire immediately after the 8 h post-instillation assessment for each study drug. The questionnaire evaluated ocular irritation, blurred vision, conjunctival hyperemia, and bitterness based on known ocular side effects associated with the study medications. Safety-related adverse events were monitored throughout the study period until study completion.

### 2.9. Primary Outcome Measure

The primary outcome was the change in the aqueous column width in the episcleral veins 2 h after instillation of each study drug. The primary comparisons focused on the differences between GLAALPHA and each of the other study medications.

### 2.10. Secondary Outcome Measures

Secondary outcomes included changes in aqueous column width at 8 h after instillation, aqueous humor flow rate in episcleral veins assessed using hemoglobin video imaging, changes in IOP at 2 and 8 h after instillation, safety and tolerability outcomes assessed using participant-reported questionnaires, and the incidence of adverse events.

### 2.11. Sample Size

Because variance estimates for between-treatment differences were not available from previous studies, a conservative assumption of zero covariance among treatments was used for the sample size estimation. Based on our previous study [18], ripasudil increased aqueous column width by +28.8% (standard deviation [SD], 16.3%), whereas latanoprost showed a change of −2.5% (SD, 5.7%), corresponding to a between-treatment difference of 31.3%.

As no prior data were available for GLAALPHA, Ailamide, or Aiphagan, expected values were determined based on their pharmacological profiles. The expected percentage changes were set at +28.8% (SD, 16.3%) for GLAALPHA, −2.5% (SD, 5.7%) for Ailamide, and −1.25% (SD, 5.7%) for Aiphagan.

Because both GLAALPHA and Ailamide contain brimonidine tartrate, which suppresses aqueous humor production and may attenuate differences in episcleral venous outflow, and given the uncertainty in extrapolating from prior studies, the expected between-treatment differences were conservatively assumed to be approximately half of the previously observed difference (15.7% for GLAALPHA vs. Ailamide and 14.4% for GLAALPHA vs. Aiphagan).

Using a fixed-sequence testing procedure to control for multiplicity, a total sample size of 24 participants (four participants per sequence across six sequences) was estimated to provide greater than 90% power to detect statistically significant differences for both primary comparisons at a two-sided significance level of 0.05.

### 2.12. Statistical Analysis

Efficacy analyses were conducted using a linear mixed-effects model, with treatment, period, and sequence included as fixed effects and participants included as a random effect. A fixed-sequence testing procedure was specified in the study protocol to control for multiplicity during the primary endpoint analysis. First, the difference in the primary outcome (percentage change in aqueous column width 2 h after instillation) between GLAALPHA and Ailamide was evaluated at a two-sided significance level of 5%. Only if this comparison reached statistical significance, the subsequent comparison between GLAALPHA and Aiphagan was performed at the same two-sided significance level of 5%. Changes in the aqueous column width and IOP before and 2 and 8 h after instillation were analyzed using a paired *t*-test. Safety and tolerability outcomes were analyzed using the exact Kruskal–Wallis test due to the non-normal distribution of the data. Data were presented as mean ± SD unless otherwise specified. All statistical analyses were performed using the appropriate statistical software.

## 3. Results

### 3.1. Participant Flow and Baseline Characteristics

**Figure 1:**
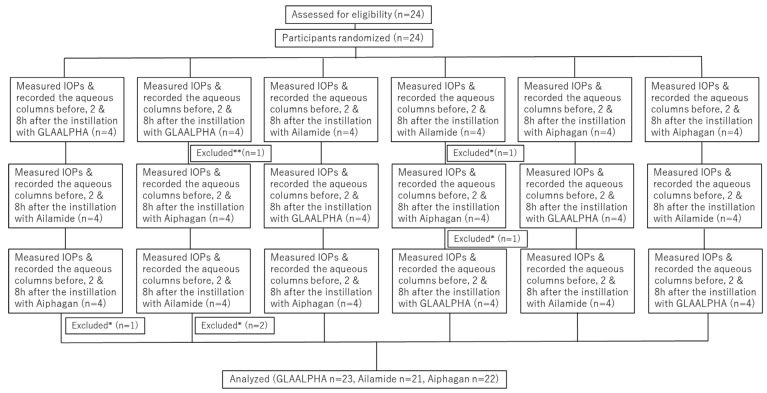
Flowchart of participant progress in this study. IOP, intraocular pressure. * Excluded due to marked blanching of episcleral veins after instillation. ** Excluded due to missing episcleral vein measurement.

A total of 24 participants were assessed for eligibility and randomized into a crossover study. Five measurements were excluded during the episcleral vein imaging procedure because reliable images could not be obtained. These were excluded because the vessel wall was indistinct and could not be clearly delineated at baseline (corresponding to “Excluded*” in Figure 1). Following instillation, some eyes became unmeasurable due to vascular blanching: unmeasurable measurements were observed after Aiphagan instillation in 8 eyes at 2 h and 4 eyes at 8 h and after Ailamide instillation in 5 eyes at 2 h and 4 eyes at 8 h. No unmeasurable cases were observed 2 h after GLAALPHA instillation, whereas 2 eyes were not measurable at 8 h. These images show marked blanching of the episcleral veins after instillation, which was considered most likely to represent a pharmacological response to brimonidine tartrate, resulting in an inability to delineate the aqueous column accurately. When post-instillation measurements were not measurable owing to vascular blanching, missing values were imputed using the last observation carried forward (LOCF) approach: missing 2 h values were replaced with the corresponding pre-instillation (baseline) values, and missing 8 h values were replaced with the corresponding 2 h values. In the sensitive analysis excluding missing values, the *p*-value of the difference between drugs using a linear mixed model was 0.040. Even without handling missing data with LOCF, similar results were obtained, suggesting that the missing data treatment did not substantially bias the results. In addition, 1 patient had missing episcleral vein measurements and was excluded from the primary outcome analysis (corresponding to “Excluded**” in Figure 1).

As a result of these exclusions, the number of eyes included in the per-protocol analyses differed slightly among treatments (GLAALPHA, *n* = 23; Ailamide, *n* = 21; Aiphagan, *n* = 22). The participant flow diagram is shown in Figure 1.

The baseline demographic and ocular characteristics are summarized in Table 1. There were no statistically significant differences among the treatment sequences with respect to age, sex distribution, eye laterality, baseline IOP, or location of the episcleral veins selected for imaging, maintaining balance among the treatment conditions.

### 3.2. Changes in Aqueous Column Width

**Figure 2:**
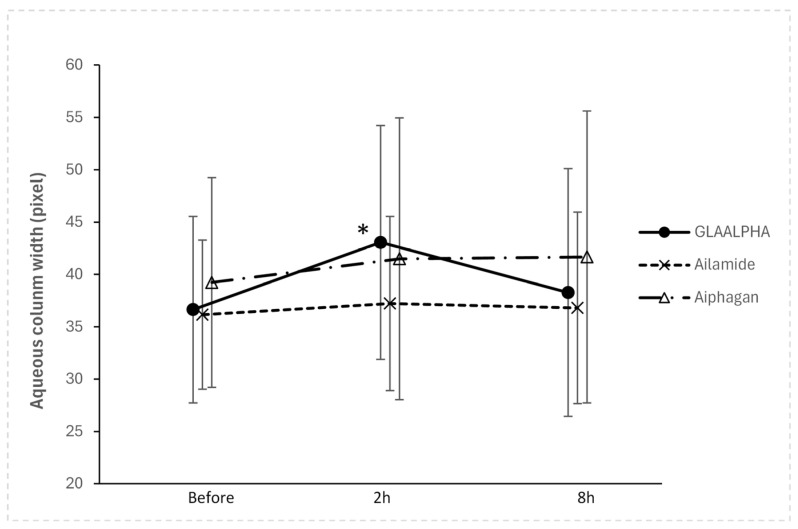
Mean change in aqueous column width before and after the instillation of each drug. At 2 h after instillation, the aqueous column width increased significantly from baseline in the GLAALPHA group (*p* = 0.002; paired *t* test). In contrast, no significant change from baseline was observed in either the Ailamide or Aiphagan groups at the same time point. * Significant difference from baseline in the GLAALPHA group (*p* = 0.002; paired *t* test).

Two hours after instillation, the aqueous column width increased significantly from the baseline in the GLAALPHA group (*p* = 0.002; paired *t*-test). Representative videos obtained before GLAALPHA instillation and 2 h after instillation are shown (Supplementary Videos S1 and S2, respectively). In contrast, no significant changes from baseline were observed in either the Ailamide (*p* = 0.305) or Aiphagan (*p* = 0.167) groups at the same time point (Figure 2).

Table 2 presents the absolute pixel values and percent changes in the aqueous column width before and after the instillation of each study drug. Regarding the primary outcome, between-group comparisons at 2 h after instillation demonstrated that the percent increase in the aqueous column width was significantly greater with GLAALPHA than with Ailamide (*p* = 0.039; linear mixed model). Although the aqueous column width tended to be larger with GLAALPHA than with Aiphagan, the difference was not statistically significant (*p* = 0.114).

**Figure 3:**
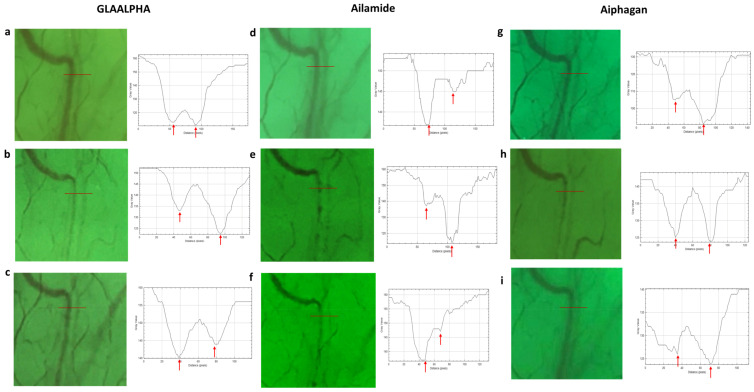
Graph of a representative case with GLAALPHA, Ailamide, and Aiphagan instillations: (**a**) before GLAALPHA instillation; (**b**) 2 h after GLAALPHA instillation; (**c**) 8 h after GLAALPHA instillation; (**d**) before Ailamide instillation; (**e**) 2 h after Ailamide instillation; (**f**) 8 h after Ailamide instillation; (**g**) before Aiphagan instillation; (**h**) 2 h after Aiphagan instillation; and (**i**) 8 h after Aiphagan instillation. The line presents the measurement position. The arrow presents the minimum intensity.

Eight hours after instillation, the aqueous column width in the GLAALPHA group showed a tendency to return to baseline values, with no statistically significant difference compared with baseline (*p* = 0.273). Similarly, no significant changes in the aqueous column width were observed 8 h after instillation in either the Ailamide (*p* = 0.548) or Aiphagan (*p* = 0.137) groups. A representative case is shown in Figure 3.

In the linear mixed-effects model analysis, neither treatment sequence nor period showed statistically significant effects (sequence: *p* = 0.736; period: *p* = 0.489), suggesting that carryover and period effects were unlikely to have influenced the study outcomes.

### 3.3. Intraocular Pressure

The changes in IOP are summarized in Table 3. IOP decreased significantly from baseline at both 2 and 8 h after the instillation of all three study drugs. At the 2 h time point, IOP was significantly lower with GLAALPHA than with Aiphagan (*p* = 0.037; linear mixed model). No significant differences were observed between GLAALPHA and Ailamide, or between Ailamide and Aiphagan. At 8 h, the magnitude of the IOP reduction did not differ among the three ophthalmic solutions.

### 3.4. Aqueous Humor Flow Rate

**Figure 4:**
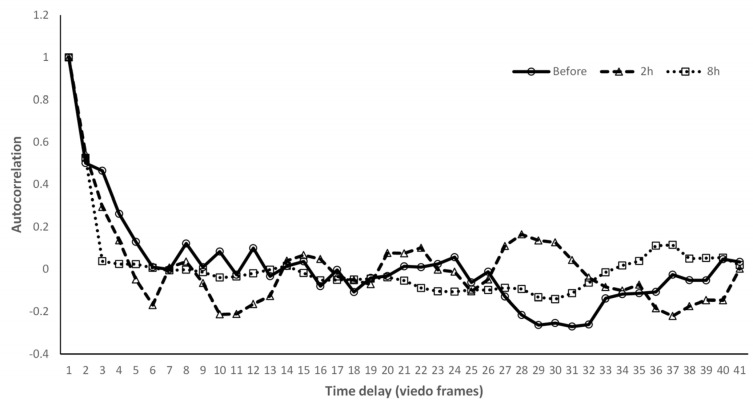
Graph of a representative case of flow rate before and after the instillations of GLAALPHA. Flow rate did not change after instillation compared with baseline.

As a secondary outcome, aqueous humor flow rates in the episcleral veins were evaluated using hemoglobin video imaging. The flow rates at baseline and at 2 and 8 h after instillation of the three study drugs were plotted for each participant. The graph in Figure 4 illustrates the speed of red blood cell flow within the episcleral veins.

Across all time points examined, no marked or consistent differences in flow rates were observed among GLAALPHA, Ailamide, and Aiphagan. In addition, no clear time-dependent trends in flow rate were detected following instillation of any of the study medications.

### 3.5. Comfort and Tolerability

**Figure 5:**
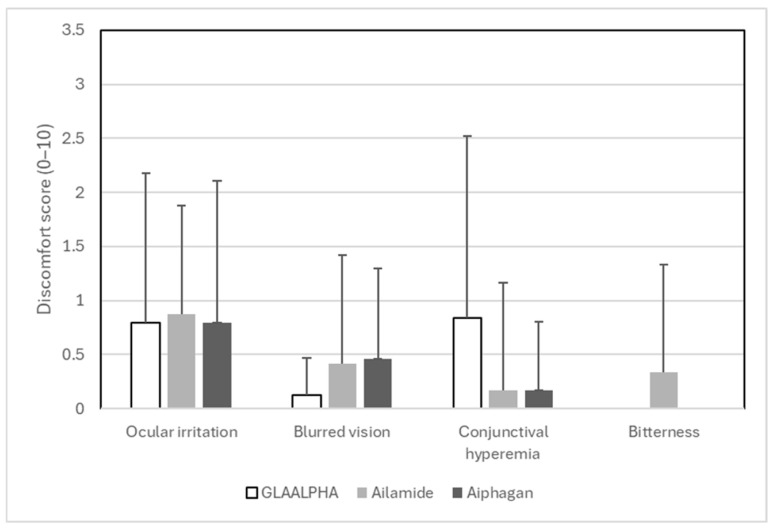
Comparison of discomfort scores among GLAALPHA, Ailamide, and Aiphagan. The frequencies of ocular irritation (*p* = 0.809), blurred vision (*p* = 0.232), conjunctival hyperemia (*p* = 0.088), or bitterness (*p* = 0.102) did not differ significantly among the three study medications.

The subjective comfort outcomes are summarized in Figure 5. The frequencies of ocular irritation, blurred vision, conjunctival hyperemia, and bitterness were generally low and did not differ significantly on the use of the three study medications. Overall, the subjective tolerability profiles of GLAALPHA, Ailamide, and Aiphagan were comparable.

### 3.6. Safety

Safety was assessed throughout the study period. No adverse events associated with any medications were observed. No cases of blepharitis, allergic conjunctivitis, keratitis, iritis, visual disturbances, or systemic adverse reactions occurred during the study period.

## 4. Discussion

The present randomized, double-masked crossover clinical trial (the ROCK Alpha-Aqua study) was designed to determine whether GLAALPHA enhances conventional aqueous humor outflow in vivo, assessed using changes in aqueous column width within the episcleral veins measured using hemoglobin video imaging. The principal finding of this study was that GLAALPHA induced a significant increase in the aqueous column width 2 h after instillation (*p* = 0.002), whereas no significant changes were observed with either Ailamide or Aiphagan. This enhancement of the aqueous column was transient and was not sustained at 8 h, which is consistent with the known pharmacodynamic profile of ripasudil hydrochloride hydrate [14]. At 2 h after instillation, the percentage increase in the aqueous column width was significantly greater with GLAALPHA than with Ailamide (*p* = 0.039). Collectively, these findings indicate that GLAALPHA promotes conventional aqueous outflow, providing direct in vivo mechanistic evidence for the role of ripasudil hydrochloride hydrate in this fixed-dose combination.

Previously, it was unclear whether the addition of a ROCK inhibitor to a brimonidine tartrate-containing ophthalmic solution would result in a measurable enhancement of conventional aqueous outflow at the level of the episcleral veins in humans. Brimonidine tartrate is known to lower IOP predominantly through the suppression of aqueous humor production and the facilitation of uveoscleral outflow, whereas its effects on the conventional pathway are considered minimal [23]. Although our previous clinical study demonstrated that ripasudil hydrochloride hydrate alone increases the aqueous column width in the episcleral veins [18], no study has directly compared a ripasudil hydrochloride hydrate-containing fixed-dose combination with brimonidine tartrate-containing ophthalmic solutions under identical experimental conditions.

The present ROCK Alpha-Aqua study is unique in that it directly visualized and quantified the conventional outflow responses to GLAALPHA in vivo. Importantly, this study demonstrated that the conventional outflow-enhancing effect of ripasudil hydrochloride hydrate is preserved within a fixed-dose combination that also contains an anti-glaucomatous agent that suppresses aqueous humor production and enhances uveoscleral outflow. Notably, a significant difference in the aqueous column width 2 h after instillation was observed between GLAALPHA and Ailamide, whereas no such difference was detected between GLAALPHA and Aiphagan. Because brimonidine tartrate was a common component across all treatment arms, this between-group difference was most plausibly attributable to the distinct pharmacological actions of ripasudil hydrochloride hydrate and brinzolamide. Ripasudil hydrochloride hydrate exerts vasodilatory effects on the distal outflow structures, including the episcleral veins, thereby increasing the capacity of the conventional outflow pathway, whereas brinzolamide primarily reduces aqueous humor production. In healthy eyes, a reduction in aqueous humor formation by brinzolamide may lead to a relative decrease in the aqueous volume entering the episcleral venous system, which could attenuate the measurable widening of the aqueous column. These opposing mechanisms, vasodilation and outflow facilitation with ripasudil hydrochloride hydrate versus aqueous suppression with brinzolamide, may explain why a significant difference in aqueous column width emerged specifically between GLAALPHA and Ailamide at the 2 h time point. Furthermore, this study extends the application of hemoglobin video imaging as a functional biomarker for pharmacological modulation of the aqueous outflow system. The present study reinforces its utility beyond surgical interventions, such as trabecular bypass stents or laser trabeculoplasty.

Despite significant widening of the aqueous column, the aqueous humor flow rate did not increase following GLAALPHA instillation. This finding contrasts with the observations following selective laser trabeculoplasty, in which both the aqueous column width and flow rate increased [20]. A plausible explanation is that ROCK inhibitors exert a vasodilatory effect on episcleral veins, as demonstrated in animal [8,25] and ex vivo [9] studies, leading to the expansion of the vascular cross-sectional area. Such dilation would increase the capacity of the vessel to accommodate the aqueous humor without necessarily increasing the linear flow velocity, thereby explaining the dissociation between the column width and flow rate.

In some participants, the aqueous column width could not be reliably measured after the instillation of brimonidine tartrate-containing formulations because of marked blanching of the episcleral veins. This phenomenon was observed not only with the fixed-dose combination but also with brimonidine tartrate monotherapy, strongly suggesting a causal relationship with brimonidine tartrate-induced vasoconstriction. Brimonidine tartrate is known to cause conjunctival blanching as an adverse pharmacological reaction [26], and the present findings indicate that this vasoconstrictive response extends to the episcleral veins, thereby precluding the accurate measurement of aqueous columns. Representative videos obtained before Aiphagan instillation and 2 h after instillation are shown (Supplementary Videos S3 and S4, respectively), with vascular blanching evident in the post-instillation video.

Although IOP decreased significantly after the instillation of all three study drugs, the magnitude of IOP reduction differed among treatments. Two hours after instillation, IOP was significantly lower with GLAALPHA than with Aiphagan, indicating an additive IOP-lowering effect of the ripasudil hydrochloride hydrate–brimonidine tartrate fixed-dose combination beyond that achieved with brimonidine tartrate alone [27]. In contrast, no significant difference in IOP reduction was observed between the Ailamide and Aiphagan groups. This finding is likely attributable to the relatively modest IOP-lowering efficacy of brinzolamide, particularly under the conditions of the present study. Because the participants were healthy volunteers with a low baseline IOP, the additional IOP reduction achievable with a carbonic anhydrase inhibitor may have been limited and insufficient to reach statistical significance within this sample size [28]. Furthermore, the present study included only 24 participants, whereas pivotal glaucoma clinical trials typically enroll more than 100 patients per treatment arm [16,29], suggesting limited statistical power to detect small between-treatment differences.

The aqueous column response to GLAALPHA exhibited a clear time dependency, with a significant increase at 2 h but not at 8 h after instillation. GLAALPHA is designed for twice-daily dosing, and it is well-established that the peak IOP-lowering effect of ripasudil hydrochloride hydrate occurs approximately 2 h after instillation [15,30]. The consistency between these known pharmacodynamic properties and the present findings suggests that the increase in the aqueous column width in the episcleral veins is closely related to the peak IOP-lowering effects of GLAALPHA.

In terms of ocular comfort, no statistically significant differences were observed the three study drugs for any of the assessed symptoms. The relatively high frequency of burning sensations across all treatments may be attributable to the fact that the participants were healthy volunteers who received anti-glaucoma eye drops for the first time and may therefore have been more sensitive to ocular surface sensations than glaucomatous patients receiving chronic topical therapy. Blurred vision is a well-recognized adverse effect of suspension formulations, such as Ailamide [31]; however, in the present study, only a small number of participants reported blurred vision after Ailamide instillation. This may be explained by the preserved visual function in healthy subjects, in whom the transient optical disturbance caused by suspension particles may not have been sufficiently pronounced to be perceived as clinically relevant. Conjunctival hyperemia, a known pharmacological effect of ripasudil hydrochloride hydrate [28,32], was observed in approximately 30% of the participants receiving GLAALPHA. Similarly, bitterness, a characteristic adverse effect associated with brinzolamide, was observed in 12.5% of the participants treated with Ailamide [33]. Overall, these comfort profiles were consistent with the known safety characteristics of each drug component.

This study has several limitations. First, the study was conducted in healthy adult volunteers rather than in patients with glaucoma. The structure and function of the trabecular meshwork and the distal outflow system may differ substantially in glaucomatous eyes, potentially attenuating or altering their responses to ROCK inhibitors. In glaucomatous eyes, pathological changes in the trabecular meshwork including extracellular matrix deposition, tissue stiffness, and cellular dysfunction may affect the response to ROCK inhibitors. In addition, structural and functional alterations in the distal outflow pathways such as collector channels and episcleral veins may further disturb aqueous outflow and the drug response. Therefore, the drug effect in healthy eyes might not completely reflect those in glaucomatous eyes. Second, the aqueous column measurements were limited to a single episcleral vein per eye, which may not fully represent the global aqueous outflow through the entire conventional pathway. Third, because the measurements were restricted to a single instillation, the effects of repeated instillations over a longer period remain unknown. A long-term study is required to determine whether GLAALPHA provides sustained enhancement of the aqueous column width in the episcleral veins.

In conclusion, the ROCK Alpha-Aqua study demonstrated that GLAALPHA significantly increased the aqueous column width in the episcleral veins 2 h after instillation in healthy adult eyes. This finding supports the concept that a ROCK inhibitor-containing fixed-dose combination can improve aqueous outflow facilities in the conventional outflow pathway, even when combined with agents that lower IOP through different pharmacological mechanisms. These findings suggest that GLAALPHA is a useful therapeutic option for enhancing conventional aqueous outflow in glaucoma management, although further studies in glaucomatous eyes and under long-term treatment conditions are required.

## Figures and Tables

**Table 1:** Baseline characteristics.

	Aiphagan-Ailamide-GLAALPHA	Aiphagan-GLAALPHA-Ailamide	Ailamide-Aiphagan- GLAALPHA	Ailamide-GLAALPHA-Aiphagan	GLAALPHA-Aiphagan- Ailamide	GLAALPHA-Ailamide-Aiphagan	*p* Value
Male. eyes (%)	2 (50%)	3 (75%)	2 (50%)	4 (100%)	3 (75%)	1 (25%)	0.529 *
Age, years mean (SD)	35.5 (19.4)	24.5 (5.2)	32.8 (18.9)	22.3 (1.7)	42.5 (23.1)	29.8 (8.7)	0.348 **
Right, eyes (%)	3 (75%)	4 (100%)	2 (50%)	2 (50%)	3 (75%)	2 (50%)	0.586 *
Baseline IOP mmHg, mean (SD)	13.5 (3.1)	13.0 (0.8)	13.5 (2.4)	11.0 (2.4)	13.8 (3.0)	13.0 (0.8)	0.585 ***
The aqueous veins in inferonasal, eyes (%)	4 (100%)	4 (100%)	3 (75%)	4 (100%)	3 (75%)	4 (100%)	1.000 *

* Fisher’s exact test. ** Welchs ANOVA. *** ANOVA.

**Table 2:** Changes in aqueous column width after instillation of GLAALPHA, Ailamide, and Aiphagan. Descriptive values are shown in (A), and within-group and between-group statistical comparisons are shown in (B).

A. Descriptive Data					
Treatment	Baseline Width (pixels)	2 h Width (pixels)	8 h Width (pixels)	2 h % Change from Baseline	8 h % Change from Baseline
GLAALPHA	36.6 ± 8.9	43.1 ± 11.2	38.3 ± 11.8	20.4 ± 28.5	6.0 ± 25.7
Ailamide	36.2 ± 7.1	37.2 ± 8.3	36.8 ± 9.1	4.3 ± 18.8	3.0 ± 22.6
Aiphagan	39.2 ± 10.0	41.5 ± 13.5	41.7 ± 13.9	7.3 ± 24.0	7.7 ± 23.3
**B. Statistical Comparisons**					
Comparison		2 h		8 h	
Within-group comparison to baseline					
GLAALPHA		*p* = 0.002 *		*p* = 0.273	
Ailamide		*p* = 0.305		*p* = 0.548	
Aiphagan		*p* = 0.167		*p* = 0.137	
Between-group comparison of % change from baseline					
GLAALPHA vs. Ailamide		*p* = 0.039 †		—	
GLAALPHA vs. Aiphagan		*p* = 0.114		—	

Values are presented as mean ± SD. * Within-group comparison to baseline. † Between-group comparison.

**Table 3:** Intraocular pressures before and after the administration of GLAALPHA, Ailamide, and Aiphagan. Intraocular pressure is mean ± standard deviation.

	Baseline	2 h	8 h
GLAALPHA (mm Hg)	12.4 ± 2.3	9.5 ± 1.7	11.0 ± 1.9
*p* value *		*p* < 0.001 *	*p* = 0.001 *
Ailamide (mm Hg)	12.4 ± 2.2	10.6 ± 2.0	10.9 ± 2.4
*p* value *		*p* < 0.001 *	*p* = 0.004 *
Aiphagan (mm Hg)	12.4 ± 2.5	10.8 ± 2.2	10.8 ± 2.4
*p* value *		*p* = 0.002 *	*p* = 0.001 *
Absolute change in width between GLAAPHA vs. Ailamide		*p* = 0.131	*p* = 0.976
Absolute change in width between GLAAPHA vs. Aiphagan †		*p* = 0.037 †	*p* = 0.976
Absolute change in width between Ailamide vs. Aiphagan		*p* = 0.835	*p* = 1.000

* Within-group comparison to baseline. † Between group comparison.

## Data Availability

The data that support the findings of this study are available from the corresponding author, upon reasonable request.

## Supplementary Materials

The following supporting information can be downloaded at: https://www.mdpi.com/article/10.3390/jcm15082880/s1, Videos S1–S4.

## Abbreviations

The following abbreviations are used in this manuscript:

IOP	intraocular pressure
ROCK	Rho-associated protein kinase
GLAALPHA	fixed-dose combination of ripasudil hydrochloride hydrate 0.4% and brimonidine tartrate 0.1%
SD	standard deviation
